# Retinoic acid induces NELFA‐mediated 2C‐like state of mouse embryonic stem cells associates with epigenetic modifications and metabolic processes in chemically defined media

**DOI:** 10.1111/cpr.13049

**Published:** 2021-05-07

**Authors:** Yanqiu Wang, Qin Na, Xihe Li, Wee‐Wei Tee, Baojiang Wu, Siqin Bao

**Affiliations:** ^1^ The State Key Laboratory of Reproductive Regulation and Breeding of Grassland Livestock Inner Mongolia University Hohhot China; ^2^ Research Center for Animal Genetic Resources of Mongolia Plateau, College of Life Sciences Inner Mongolia University Hohhot China; ^3^ Basic Medical College Inner Mongolia Medical University Hohhot China; ^4^ Inner Mongolia Saikexing Institute of Breeding and Reproductive Biotechnology in Domestic Animal Huhhot China; ^5^ Chromatin Dynamics and Disease Epigenetics Group Institute of Molecular and Cell Biology Agency for Science, Technology and Research (A*STAR) Singapore Singapore; ^6^ Department of Physiology Yong Loo Lin School of Medicine, National University of Singapore Singapore Singapore

**Keywords:** 2C‐like cells, embryonic stem cell, epigenetic, mouse, NELFA, retinoic acid

## Abstract

**Objectives:**

Mouse embryonic stem cells (ESCs) are derived from the inner cell mass of blastocyst‐stage embryos and cultured in different culture media with varied pluripotency. Sporadically, a small population of ESCs exhibit 2‐cell stage embryonic features in serum containing medium. However, whether ESCs can transit into 2‐cell embryo‐like (2C‐like) cells in the chemically defined media remains largely unknown.

**Materials and Methods:**

We established a robust in vitro induction system, based on retinoic acid (RA) containing chemically defined media, which can efficiently increase the subpopulation of 2C‐like cells. Further test the pluripotency and 2C features of ESCs cultured in RA. 2C reporter‐positive cells were selected by FACS; the level of protein was detected via immunofluorescence staining and western blot; the level gene expressions were measured by RNA‐seq.

**Results:**

Retinoic acid drives a NELFA (negative elongation factor A)‐mediated 2C‐like state in mouse ESCs, characterized with 2C‐specific transcriptional networks and the ability to contribute trophectoderm (TE) when injected into developing embryos. In addition, RA treatment triggers DNA hypomethylation, active histone modification, suppressed glycolysis metabolism and reduced protein synthesis activity of ESCs.

**Conclusions:**

We showed that RA has a broader role in 2C‐like cells state, not only is one of the upstream regulators of the 2C‐like state in chemically defined media but also illuminates genetic and epigenetic regulations that govern ESCs to 2C‐like transition.

## Graphical abstract

2C‐like cells arise spontaneously in ESCs upon serum and leukemia inhibitory factor (LIF) containing culture medium and 2C‐like cells refers to totipotent. Yet, the establishment of totipotency is poorly understood. Intriguingly, naïve ESCs culture medium fail to induce 2C‐like state of ESCs. In this study, we report a robust in vitro induction system, based on retinoic acid (RA) containing chemically defined media, which can efficiently increase 2C‐like cells population in culture without any genetic modification and serum. As ever more mechanisms of totipotent cells are unraveled, data of this study will provide new impulses for stem cell research.

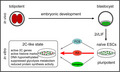

## 1 INTRODUCTION

Embryonic stem cells are thought of as a budding of early developmental stage embryos that retain the capacity to differentiate into nearly all cell types except extra‐embryonic tissues in chimeric embryos.^1^, ^2^ In addition, in vitro cultured ESCs exhibit heterogeneous and retain small populations of cells harbours totipotent which expressing a set of genes are active in 2‐cell stage embryos.^3^, ^4^, ^5^ These 2‐cell‐like (2C‐like) cells display several features of 2‐cell embryos, including expression of 2‐cell embryo specific transcripts, active histone modifications, DNA hypomethylation, and the capacity to contribute to both inner cell mass (ICM) and trophectoderm (TE).^6^ In mice, the zygotic genome is activated at 2‐cell stage by a process known as zygotic genome activation (ZGA) and followed by the gain of totipotency.^7^ Thus, 2C‐like state in ESCs is an invaluable tool to address the mechanisms underlying 2‐cell stage embryonic development.

2‐cell embryo‐like cells arise spontaneously in serum and leukaemia inhibitory factor (LIF) containing culture condition (S/L) and regulated by multiple factors, including zinc‐finger and SCAN domain containing 4 (*Zscan4*),^8^ mouse endogenous retrovirus with leucine tRNA primer (MERVL),^3^ the transcriptional factor double homeobox (*Dux*),^9^ negative elongation factor complex member A (NELFA)^10^ and *Duxbl1* ^11^ as well as developmental pluripotency‐associated 2 (*Dppa2*) and *Dppa4*.^12^, ^13^ Furthermore, previous studies indicated that 2C‐like state is suppressed by ZGA repressor, such as tripartite motif‐containing protein 28 (TRIM28; also known as KAP1),^14^ long interspersed nuclear element‐1 (LINE1),^14^ lysine (K)‐specific demethylase 1A (LSD1/KDM1A),^15^ chromatin assembly factor‐1 (CAF‐1)^16^ and microRNA *miR‐34a*.^17^ In addition, these 2C‐like cells spontaneously transit back into the pluripotent state.^18^ This showed that ESCs and 2C‐like cells are in keeping with a dynamic equilibrium state.

2‐cell embryo‐like cells of ESCs are induced in S/L medium, but the composition of serum is more complex, which factors play a key role on 2C‐like cells activation remains largely undefined. Several studies reported that retinoic acid (RA) signalling leads to increase of 2C‐like cells in S/L cultured ESCs (S/L‐ESCs).^11^, ^19^ RA induces ESCs transition to 2C‐like state through a coordinated expression of *Dux* and *Duxbl1* or activation of prame family member *Gm12794c*.^20^ Notably, it is well known that naïve ESCs maintenance medium fails to induce 2C‐like state of ESCs.^10^ Altogether, it remains unclear whether RA has a beneficial effect on 2C‐like state of ESCs in 2i/L chemically defined media.

We previously investigated a negative elongation factor A (NELFA) whose expression in mouse ESCs is coupled to 2C‐like state and expanded fate potential in vivo.^10^ In this study, we found that RA induces 2C‐like cells in mouse ESCs that are cultured in N2B27 based 2i/L chemically defined media. Notably, we demonstrated that RA activates NELFA‐mediated 2C‐like state of mouse ESCs and upregulates the 2C‐specific genes expression in long‐term in vitro culture as well as exhibits expanded developmental potency in vivo. In addition, our results indicated that epigenetic regulations and metabolic processes play an important role in RA‐induced 2C‐like state.

## 2 MATERIALS AND METHODS

### 2.1 Mice

The mice were housed in the animal facility of Inner Mongolia University, China. All animal maintenance and experiments we performed in accordance with the guidelines of Institutional Animal Care and Use Committee, Inner Mongolia University, China. The mice were sacrificed by cervical dislocation. The 8‐cell stage embryos were collected from the oviducts of female ICR mice. To obtain chimeric embryos, 8‐cell stage embryos were cultured in KSOM for 48 hours after microinjection.

### 2.2 Embryonic stem cells culture

The MERVL::tdTomato and NELFA‐GFP reporter ESCs were cultured on fibronectin‐coated (16.7 μg/mL, Millipore) cell culture plates with N2B27 medium supplemented with 1000 IU/mL LIF, 3 µmol/L CHIR99021 and 1 µmol/L PD0325901. The following retinoic acid inducible cell lines cultured with N2B27 medium supplemented with 1000 IU/mL LIF, 3 µmol/L CHIR99021, 1 µmol/L PD0325901 and 5 µmol/L retinoic acid. N2B27 medium consisted of one volume DMEM/F12 combined with one volume Neurobasal medium supplemented with 0.5% N2 supplement, 1% B27 supplement, 2 mmol/L Glutamax, 1% MEM NEAA, 1% penicillin/streptomycin, 50 mg/L bovine serum albumin and 55 μmol/L β‐mercaptoethanol. Cells were cultured at 37°C and 5% CO_2_ and routinely passaged 1:3‐1:6 every 2 days. For inhibitor or other signalling pathway activation treatment experiment, we added Activin A (20 ng/mL), 5‐aza‐2'‐deoxycytidine (6 μmol/L), 2‐deoxy‐D‐glucose (4 mmol/L), valproic acid (10 µmol/L), scriptaid (250 nmol/L), cycloheximide (0.2 µg/mL), IWR1 (5 µmol/L) and XAV939 (10 µmol/L) into ESCs culture medium.

### 2.3 Flow cytometry

The MERVL::tdTomato and NELFA‐GFP reporter ESCs were harvested by Accutase and sorting by BD LSRFortessa. No tdTomato and GFP ESCs were used for FACS gating negative control. Measure fluorescence (detector 568 nm and 488 nm channel for tdTomato and GFP) by flow cytometer. Gating out of residual cell debris and measure diploid and tetraploid DNA peaks. A region representing tdTomato and GFP‐positive cells were used to identify living cells.

### 2.4 Real‐Time PCR (qPCR)

The cells were collected, total RNA was isolated by RNeasy Plus Mini Kit (Qiagen), and cDNA was obtained by Reverse Transcription System (Promega). qPCR reaction was performed using KAPA SYBR FAST qPCR kit (KAPA Biosystems) and repeated with at least three biological replicates with similar results. The expression levels were plotted relative to *Gapdh*. All samples were conducted using a LightCycler^®^ 96 Instrument (Roche Molecular Systems). Relative expression levels were determined by the 2^−ΔΔCt^ method. Primer sequences used are given in Table S1.

### 2.5 Western blot

Protein was extracted using lysis buffer and quantified by Pierce™ BCA Protein Assay Kit (Thermo, 23 227). 1 μL PMSF (0.1 mol/L) and 10 μL phosphatase inhibitor (10 g/L) were added per 1 mL lysis buffer immediately before use. Denatured proteins were resolved using 10%–12% SDS‐PAGE gels and blotted on PVDF membranes. Following blocking in 5% skim milk/0.2% Tween/dH_2_O, membranes were incubated with primary antibodies at 4°C for overnight. The primary antibodies used were anti‐ZSCAN4 (Abcam, ab106646; dilution 1:1000), anti‐DNMT3A (CST, 3598S; dilution 1:1000), anti‐DNMT3L (Abcam, ab3493; dilution 1:2500), anti‐DNMT1 (Abcam, ab19905; dilution 1:1000), anti‐β‐CATENIN (CST, 8480; dilution 1:1000), anti‐H3K27ac (Abcam, ab4729; dilution 1:1000), anti‐H3 (Abcam, ab176842; dilution 1:1000) and anti‐β‐ACTIN (Abcam, ab8227; dilution 1:5000). Horseradish peroxidase‐conjugated secondary antibodies (1:5000) were incubated for 1 hours at room temperature, and proteins were detected by ECL plus reagent. After rinsing with TBST, Clarity™ Western ECL Substrate (BIO‐RAD) was used for visualization, and ChemiDoc™ MP Imaging System (BIO‐RAD) was used for band detection.

### 2.6 Immunostaining

For immunofluorescence staining, the cells or pre‐implantation embryos were fixed with 4% paraformaldehyde (PFA) for 30 minutes, followed by blocking in PBS containing 1% BSA and 0.1% Triton X‐100 for 30 minutes and then stained overnight at 4°C with the primary antibody against anti‐ZSCAN4 (Abcam, ab106646, 1:500), anti‐OCT4 (BD Biosciences, 611203, 1:200) and anti‐CDX2 (BioGenes, AM392, 1:200). Next, the cells were washed three times with PBS and incubated for 1 hour at room temperature with secondary antibodies. The slides were then mounted in Vectashield with DAPI (Vector Laboratories) and imaged using an Olympus FV1000 confocal microscope.

### 2.7 RNA extraction, library construction and sequencing

Total RNA was extracted using TRIzol reagent kit (Invitrogen) according to the manufacturer's protocol. RNA quality was assessed on an Agilent 2100 Bioanalyzer (Agilent Technologies) and checked using RNase free agarose gel electrophoresis. After total RNA was extracted, eukaryotic mRNA was enriched by Oligo(dT) beads, while prokaryotic mRNA was enriched by removing rRNA by Ribo Zero^TM^ Magnetic Kit (Epicentre). Then, the enriched mRNA was fragmented into short fragments using fragmentation buffer and reverse transcripted into cDNA with random primers. Second strand cDNA was synthesized by DNA polymerase I, RNase H, dNTP and buffer. Then, the cDNA fragments were purified with QiaQuick PCR extraction kit (Qiagen), end repaired, poly (A) added and ligated to Illumina sequencing adapters. The ligation products were size selected by agarose gel electrophoresis, PCR amplified and sequenced using Illumina HiSeq 2500 by Gene Denovo Biotechnology Co.

### 2.8 RNA‐seq data analysis

For each transcription region, a FPKM (fragment per kilobase of transcript per million mapped reads) value was calculated to quantify its expression abundance and variations, using StringTie software. After removal of the reads that contained adapters, poly‐N strands and low‐quality reads, the clean data were mapped to the genome using HISAT2. The FPKM method is able to eliminate the influence of different gene lengths and sequencing data amount on the calculation of gene expression. Therefore, the calculated gene expression can be directly used for comparing the difference of gene expression among samples. StringTie software was used to calculate the expression of all genes in each sample, and DESeq2 software^21^ was used to obtain the differential genes among different groups. The threshold (false discovery rate (FDR) < .05 and the absolute value of the log2 fold change ≥1 and absolute fold change ≥2) was used for gene screening. Principal component analysis (PCA) was performed with R package models (http://www.rproject.org/) in this experience. PCA is a statistical procedure that converts hundreds of thousands of correlated variables (gene expression) into a set of values of linearly uncorrelated variables called principal components. PCA is largely used to reveal the structure/relationship of the samples/data. UHC analysis was performed by the R *hclust* function. Gene ontology (GO)^22^ is an international standardized gene functional classification system which offers a dynamic updated controlled vocabulary and a strictly defined concept to comprehensively describe properties of genes and their products in any organism. GO has three ontologies: molecular function, cellular component and biological process. The basic unit of GO is GO term. Each GO term belongs to a type of ontology. Trend analysis of DEGs was performed using Short Time‐series Expression Miner (STEM) software.^23^

### 2.9 Gene set enrichment analysis (GESA)

We performed gene set enrichment analysis using software GSEA and MSigDB^24^ to identify whether a set of genes in specific GO terms pathways. GO terms shows significant differences in two groups. Briefly, we input gene expression matrix and rank genes by Signal‐to‐Noise normalization method. Enrichment scores and *P* value were calculated in default parameters.

### 2.10 Production of chimeric embryos

6‐10 mCherry‐labelled NELFA‐positive ESCs were injected gently into the ICR mice eight‐cell stage embryos using a piezo‐assisted micromanipulator attached to an inverted microscope. The injected embryos were cultured in KSOM medium (Millipore) at 37°C in a 5% CO_2_ atmosphere for 48 hours and further performed immunostaining.

### 2.11 Statistical analysis

All values are depicted as mean ± SD. Statistical parameters including statistical analysis, statistical significance and n value are reported in the Figure legends and supporting Figure legends. Statistical analyses were performed using Prism Software (GraphPad Prism version 6). The significance of differences was measured by an unpaired two‐tailed Student's *t* test was employed. A value of *P* < .05 was considered significant.

## 3 RESULTS

### 3.1 Retinoic acid induces stable 2C‐like state of ESCs in chemically defined condition

**FIGURE 1:**
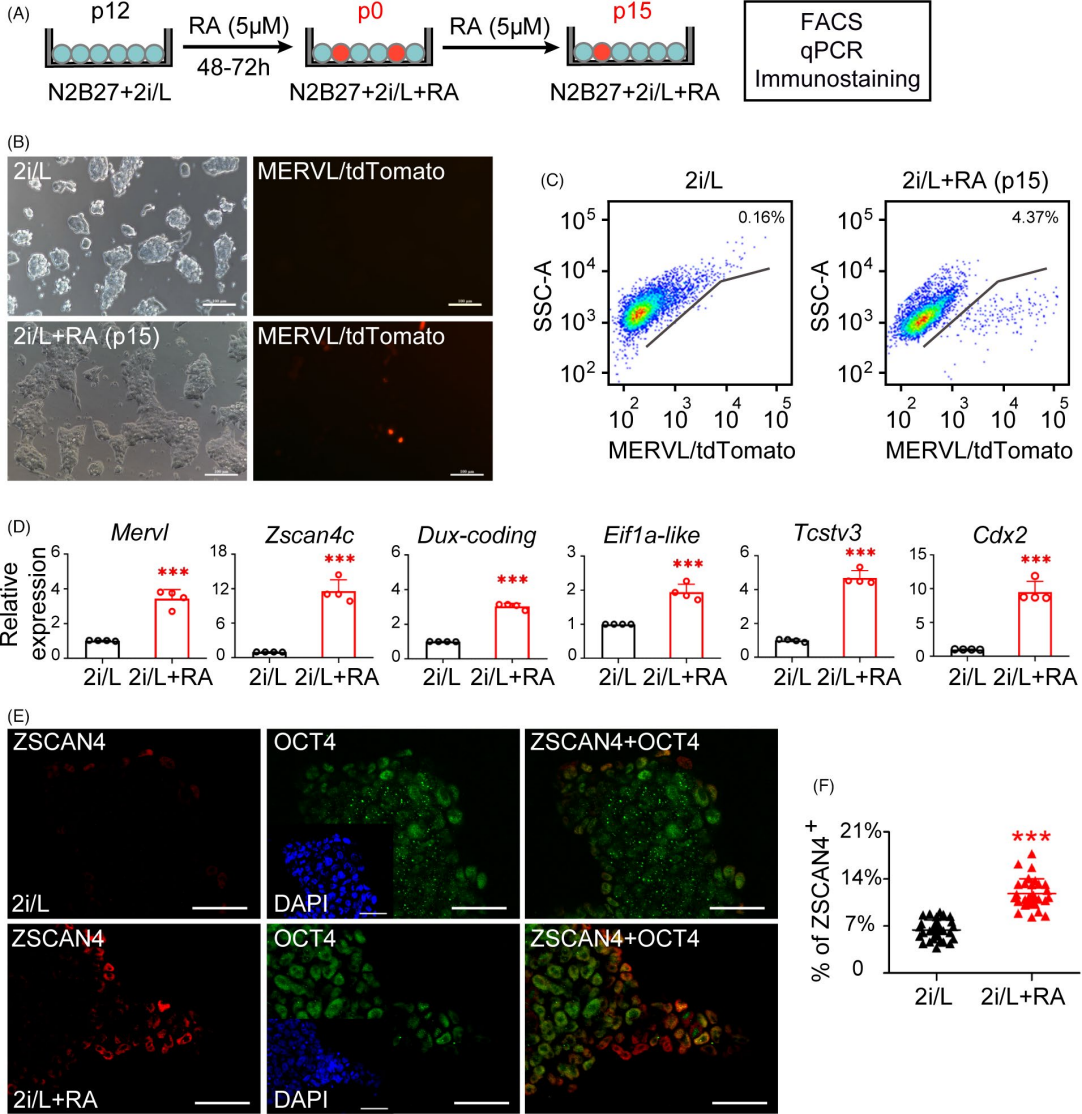
Retinoic acid induces the 2C‐like state in chemically defined media. A, Experimental outline of the MERVL::tdTomato‐labelled ESCs was treated with RA. B, MERVL::tdTomato reporter ESCs were cultured in 2i/L and 2i/L+RA medium. 2i/L+RA condition was significantly increasing the MERVL::tdTomato‐positive cells. Scale bars, 100 μm. C, tdTomato based Fluorescence‐activated cell sorting (FACS) on 2i/L and 2i/L+RA cultured MERVL::tdTomato reporter ESCs. D, Relative expression levels of 2C genes (*Mervl, Zscan4c, Dux‐coding, Eil1a‐like, Tcstv3* and *Cdx2*) measured by qPCR in 2i/L and 2i/L+RA cultured MERVL::tdTomato reporter ESCs. Error bars are mean ± SD (n = 4). *P* values were calculated by two‐tailed Student's *t* test, *P* < .05. E, Immunostaining of ZSCAN4 and OCT4 in 2i/L and 2i/L+RA cultured ESCs. Scale bars, 50 μm. F, Quantification of the ZSCAN4*‐*positive cells in 2i/L and 2i/L+RA cultured ESCs. Error bars are mean ± SD (n = 30). *P* values were calculated by two‐tailed Student's *t* test, *P* < .05

Previous studies showed that 2C‐like subpopulation could be cultured in serum and LIF condition (S/L). However, naïve culture medium (N2B27 based CHIR99021, PD0325901 and LIF; also known as 2i/L) failed to induce ESCs (2i/L‐ESCs) to a 2C‐like state.^10^ To achieve the activation of 2C‐like state under the chemically defined condition, we screened additional chemical compounds (Activin A, 2‐deoxy‐D‐glucose, 5‐aza‐2'‐deoxycytidine and valproic acid) (Figure S1A) in different basic media, and found 2C‐like cells can persist in serum plus 2i/L (S/2i/L) medium supplemented with 2DG, 5‐Aza and Scriptaid which is consistent with previous studies.^3^, ^10^ To assess whether RA regulates the MERVL::tdTomato reporter labelled 2C‐like state in serum‐free chemically defined condition, we grew passages 12 (p12) 2i/L‐ESCs (MERVL::tdTomato‐labelled) in naïve maintenance medium (2i/L) in the presence of RA (2i/L+RA) and measured the percentage of tdTomato‐positive (tdTomato^+^) cells (Figure 1A). RA treatment increase the percentage of tdTomato^+^ cells (p15) about twenty‐seven fold (4.37%) compared to without RA‐treated ESCs (0.16%) as measured by fluorescence‐activated cell sorting (FACS) (Figure 1B,C). Thus, RA can boost MERVL::tdTomato‐labelled 2C‐like cells induction in serum‐free medium. Notably, we found that levels of 2C‐like related genes (such as *Mervl*, *Zscan4c*, *Dux‐coding*, *Eif1a‐like*, *Tcstv3* and *Cdx2*) were significantly upregulated in RA‐treated 2i/L‐ESCs relative to those naïve 2i/L‐ESCs (Figure 1D). Therefore, we further asked whether RA treatment could increase the number of ZSCAN4‐positive 2C‐like cells. As expected, the number of ZSCAN4‐positive cells is increased in the presence of RA (Figure 1E,F and Figure S1B). These results indicated that long‐term RA treatment (p15) could maintain ESCs self‐renew and increase 2C‐like cells in culture.

### 3.2 Retinoic acid induces 2C‐like state of ESCs through maternal factor NELFA

**FIGURE 2:**
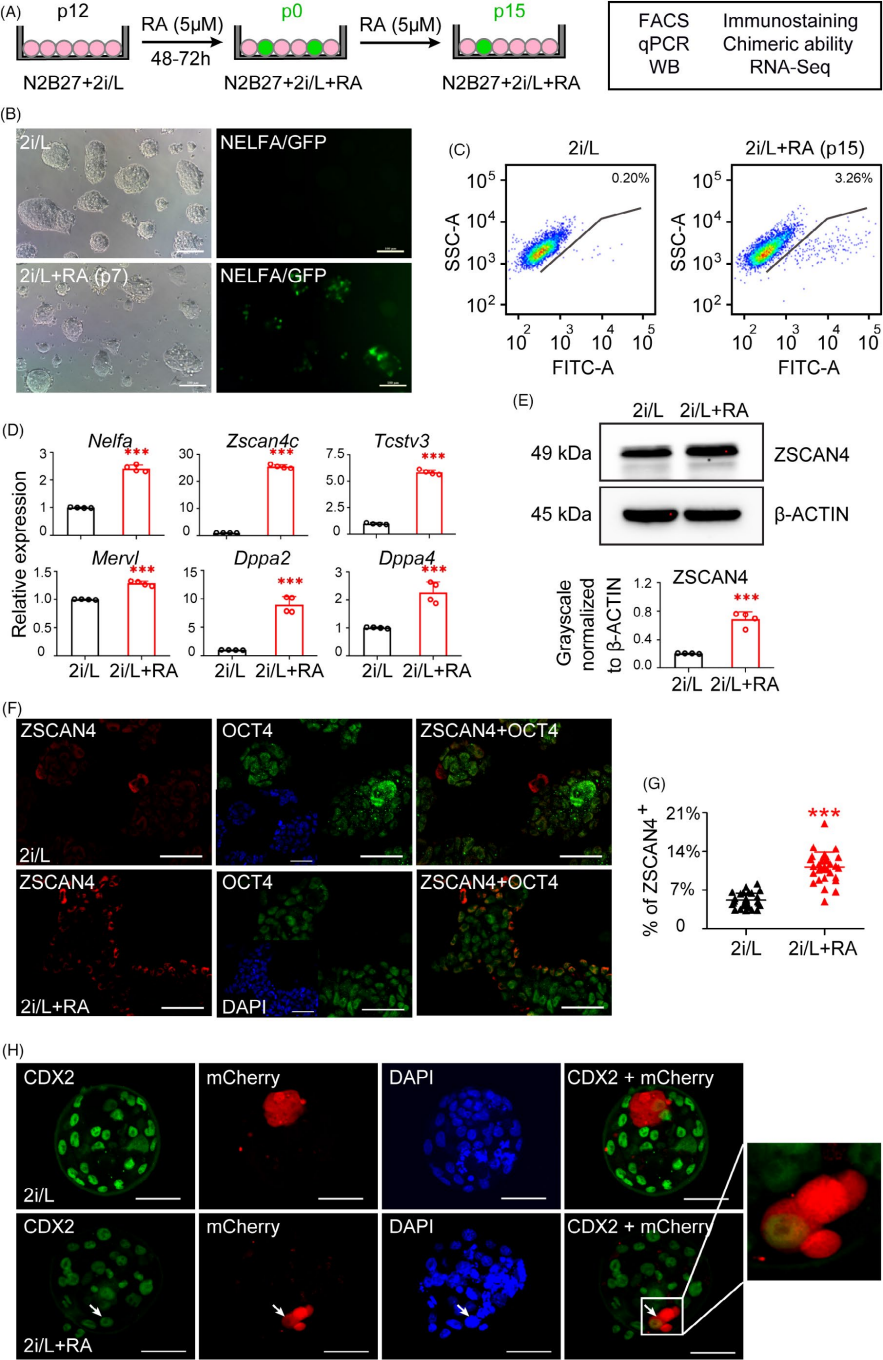
Retinoic acid drives NELFA‐mediated 2C‐like state in ESCs. A, Experimental outline of the NELFA‐GFP reporter ESCs were treated with RA. B, NELFA‐GFP reporter ESCs were cultured in 2i/L and 2i/L+RA medium. 2i/L+RA condition was significantly increasing the NELFA‐GFP‐positive cell number. Scale bars, 100 μm. C, FACS quantification of the proportion of GFP‐positive (GFP^+^) cells upon activation by RA. D, qPCR of 2C‐specific genes *Nelfa,* *Zscan4c, Tcstv3, Mervl, Dppa2* and *Dppa4* expression level in 2i/L and 2i/L+RA cultured NELFA‐GFP reporter ESCs. Error bars are mean ± SD (n = 4). *P* values were calculated by two‐tailed Student's *t* test, *P* < .05. E, Western blotting analysis for ZSCAN4 in 2i/L and 2i/L+RA cultured NELFA‐GFP reporter ESCs and the band intensity of western blotting was quantified in control. F, Immunostaining of ZSCAN4 and OCT4 in 2i/L, 2i/L+RA cultured NELFA‐GFP reporter ESCs. Scale bars, 50 μm. G, Quantification of the ZSCAN4*‐*positive cells in 2i/L and 2i/L+RA cultured ESCs. Error bars are mean ± SD (n = 30). *P* values were calculated by two‐tailed Student's *t* test, *P* < .05. H, mCherry‐labelled NELFA‐positive cells were injected into 8‐cell stage embryos, which were then cultured 48 h in vitro. mCherry‐labelled NELFA‐positive RA‐2i/L‐ESCs were able to contribute to both TE or ICM, whereas 2i/L‐ESCs were only contribute to ICM. Scale bars, 50 μm

Our previous study indicated that a *NELFA*/*Dux*/*Zscan4* regulatory axis is involved in the activation of the 2C genes in S/2i/L condition. To further investigate whether RA participates in *NELFA*/*Dux*/*Zscan4* regulatory axis, we treated the passages 12 (p12) NELFA‐GFP reporter 2i/L‐ESCs with RA (Figure 2A,B). We observed that NELFA‐GFP reporter was activated in 48 hours after RA treatment in chemically defined medium (Figure S1C). Importantly, the proportion of NELFA‐positive 2C‐like cells are increased in the presence of RA for a long term in vitro culture, over passages fifty (Figure 2C and Figure S1C). Then, we compared the efficiency of 2C‐like conversion as a function of RA treatment by fluorescence‐activated cell sorting (FACS). We also observed that upon long‐term RA treatment (p15), the NELFA‐positive cells were significantly increased when compared to no RA treatment 2i/L group (3.26% vs 0.20%, respectively) (Figure 2C). Furthermore, addition of RA in N2B27 based 2i/L medium successfully initiate 2C‐like gene expression, such as *Nelfa, Zscan4c, Tcstv3, Mervl, Dppa2* and *Dppa4* tested by quantitative real‐time PCR (qPCR) (Figure 2D). In particular, we test the protein level of ZSCAN4 by western blotting and the number of ZSCAN4‐positive cells by immunofluorescence staining. Our results indicated that the protein level of ZSCAN4 (Figure 2E) and the number of ZSCAN4‐positive cells (Figure 2F,G and Figure S1D) were significantly increased in RA‐treated 2i/L‐ESCs (RA‐2i/L‐ESCs) compared with 2i/L‐ESCs. Collectively, above results show that RA activates the *NELFA/Dux/Zscan4* regulatory axis and confirms that RA is an upstream regulator of *NELFA*/*Dux*/*Zscan4* axis.

Another most important feature of 2C‐like state is totipotency which can contribute to both inner cell mass (ICM) and trophectoderm (TE) in blastocyst. To this end, we tested the developmental potency of RA‐2i/L‐ESCs in vivo by introducing mCherry‐labelled NELFA‐positive cells into 8‐cell stage embryos, which were cultured for 48 hours in KSOM and then performed immunofluorescence staining. Remarkably, mCherry‐labelled NELFA‐positive RA‐2i/L‐ESCs were able to contribute to both embryonic inner cell mass and extra‐embryonic trophectoderm in about 19.4% (7/36) of chimeric embryos, while 2i/L‐ESCs could only contribute exclusively to the inner cell mass in all embryos analysed (0/38) (Figure 2H and Figure S1E,F). Our data showed that RA‐induced ESCs can activate the 2C‐specific genes and also can expand the cell fate determination of ESCs in chemical‐defined medium.

### 3.3 Retinoic acid regulates transcriptional networks related to 2C‐like state

**FIGURE 3:**
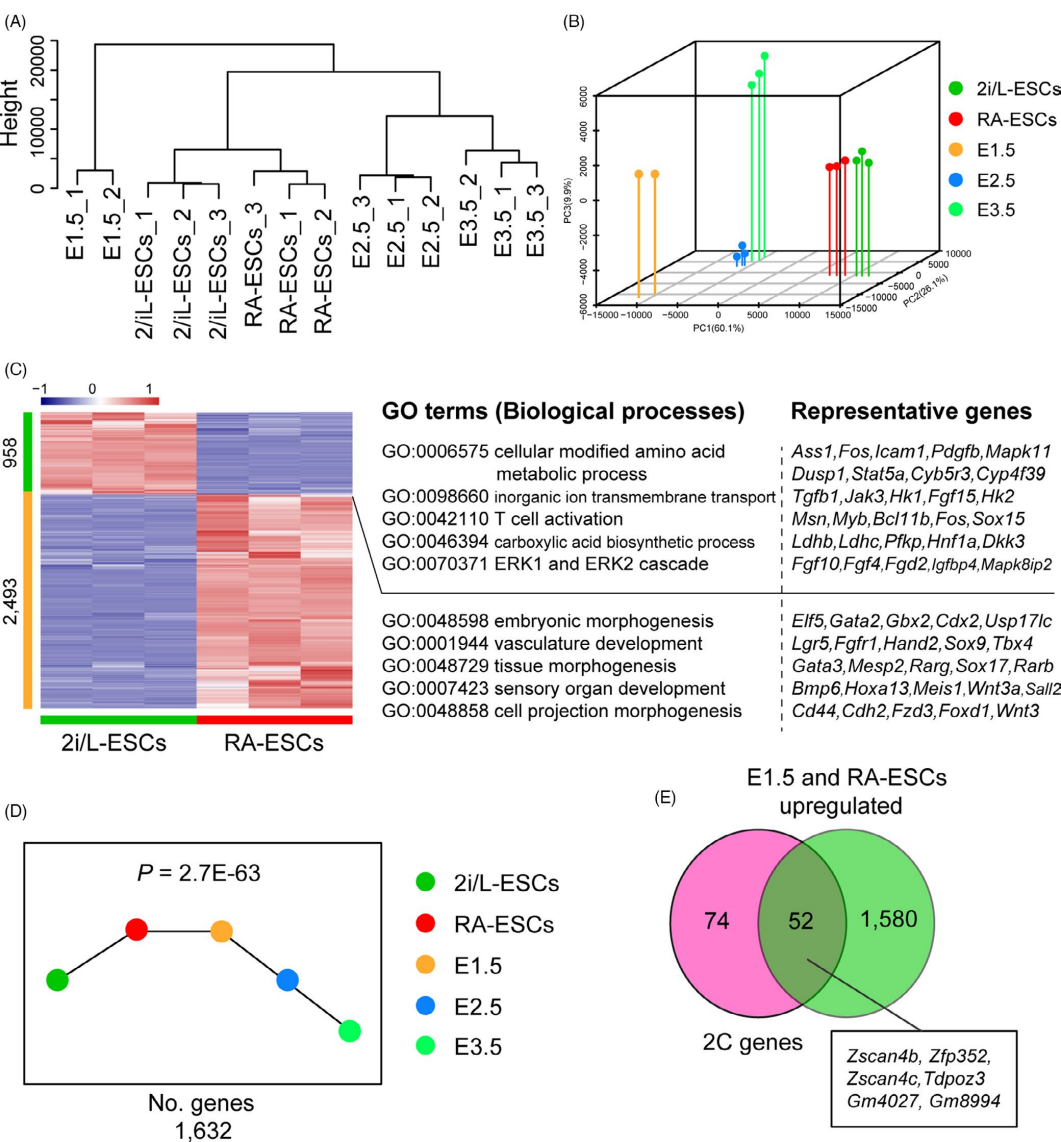
Retinoic acids regulate transcriptional networks related to 2C‐like state. A, Hierarchical clustering of transcriptomes data from pre‐implantation embryos (E1.5, E2.5 and E3.5), 2i/L‐ESCs and RA‐2i/L‐ESCs. B, Principle component analysis (PCA) of transcriptomes data from pre‐implantation embryos (E1.5, E2.5 and E3.5), 2i/L‐ESCs and RA‐2i/L‐ESCs. C, Heat map showing differentially expressed genes (mean log2 (normalized read counts) > 2, log2 (fold change) > 2, adjusted *P* value < .05) in RA‐2i/L‐ESCs compared with 2i/L‐ESCs. Significantly enriched GO terms and representative genes in each cluster are listed on the right. D, We use Short Time‐series Expression Miner (STEM) software program to analysis the gene expression trend on E1.5, E2.5, E3.5, 2i/L‐ESCs and RA‐2i/L‐ESCs. There are 1632 genes highly expressed in both RA‐2i/L‐ESCs and E1.5. E, Venn Diagram indicated that total of 52 genes were overlaped between previous reported 2C genes (126 genes) versus E1.5 and RA‐2i/L‐ESCs upregulated genes (1632 genes), such as *Zscan4b, Zfp352, Zscan4c, Tdpoz3, Gm4027* and *Gm8994*

To gain further insight into the molecular underpinnings of RA function in 2C‐like cells induction, we analyse the gene expression level of 2i/L‐ESCs (p12) and RA‐2i/L‐ESCs (p15) by RNA sequencing. Unsupervised hierarchical clustering (UHC) and principal component analysis (PCA) results of transcriptional levels on pre‐implantation embryos (E1.5, E2.5 and E3.5),^25^, ^26^ 2i/L‐ESCs and RA‐2i/L‐ESCs indicated that RA‐2i/L‐ESCs are similar to 2i/L‐ESCs (Figure 3A,B). In addition, we identified 2493 upregulated genes and 958 downregulated genes in RA‐2i/L‐ESCs compared with 2i/L‐ESCs (Figure 3C). This indicated that RA‐2i/L‐ESCs and 2i/L‐ESCs exhibit distinct transcriptional pattern. To gain further insight into the function of RA related to NELFA, we compared RA‐2i/L‐ESCs‐specific upregulated genes (2493) (RA‐2i/L‐ESCs vs 2i/L‐ESCs) with previously published NELFA‐positive upregulated genes (1086 genes) based on RNA‐seq data. We found that a total of 198 overlapping genes between RA treatment and NELFA upregulated transcripts, including most 2C‐related genes such as *Tdpoz3, Zscan4f, Usp17lc, Zscan4c, Tdpoz4* and *Tcstv3* (Figure S2A). Next, we use short time‐series expression miner (STEM) software program^23^ to analyse the gene expression trend on E1.5, E2.5, E3.5, 2i/L‐ESCs and RA‐2i/L‐ESCs. Remarkably, total of 1632 genes was significantly upregulated in both E1.5 and RA‐2i/L‐ESCs (Figure 3D). Moreover, total of 52 genes were overlap between previous reported 2C genes (126 genes) in E1.5 and RA‐2i/L‐ESCs upregulated genes (1632 genes), such as *Zscan4b, Zfp352, Zscan4c, Tdpoz3, Gm4027* and *Gm8994* (Figure 3E). This indicated that gene expression pattern of E1.5 and RA‐2i/L‐ESCs are highly correlated, especially 2C‐related genes. Importantly, gene set enrichment analysis (GSEA) also identified some upregulated gene signatures related to retinoid metabolic process in RA‐2i/L‐ESCs compared with 2i/L‐ESCs (Figure S2B,C). Thus, these results suggest that RA activates 2C genes through retinol metabolism. Taken together, our data suggest that retinoic acid regulates ESCs transcriptional networks, especially 2C related transcriptional network activation.

### 3.4 Epigenetic modifications enhance retinoic acid‐induced 2C‐like state of ESCs via NELFA

**FIGURE 4:**
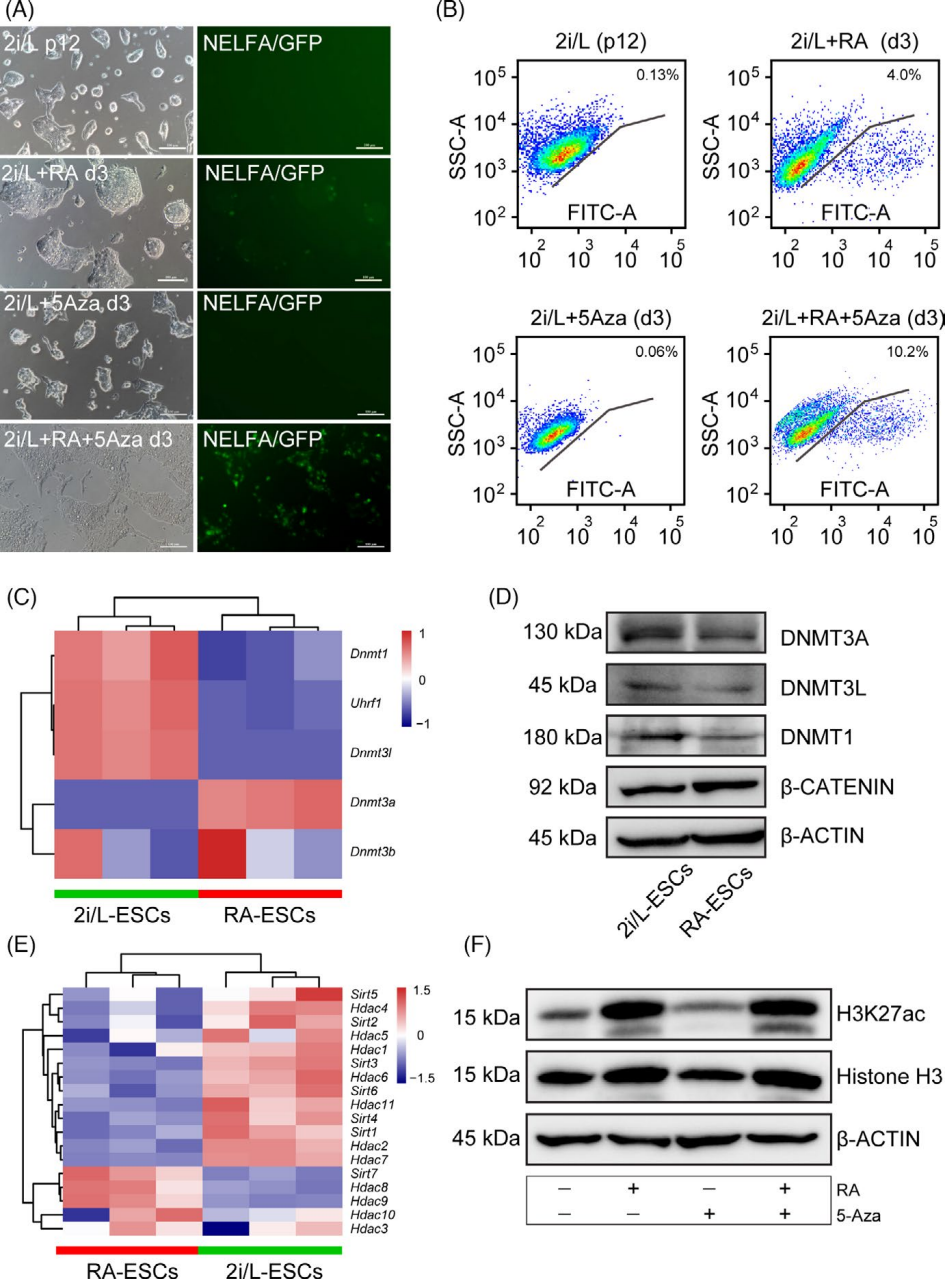
Epigenetic modifications enhance retinoic acid‐induced 2C‐like state of ESCs via NELFA. A, NELFA‐GFP reporter ESCs were cultured in 2i/L, 2i/L+RA, 2i/L+5aza and 2i/L+RA+5aza medium. 2i/L+RA+5aza condition was significantly increasing the NELFA‐GFP‐positive cell number. Scale bars, 100 μm. B, FACS quantification of the proportion of GFP‐positive (GFP^+^) cells upon activation by 2i/L, 2i/L+RA, 2i/L+5aza and 2i/L+RA+5aza. C, DNA methylation related genes heat map in 2i/L‐ESCs and RA‐2i/L‐ESCs. D, Western blotting analysis for DNMT1, DNMT3A, DNMT3L and β‐CATENIN in 2i/L‐ESCs and RA‐2i/L‐ESCs. E, Histone deacetylation related genes heat map in 2i/L‐ESCs and RA‐2i/L‐ESCs. F, Western blotting analysis for H3K37ac and histone H3 in ESCs cultured in different culture conditions

Globally, 2C‐like cells possess hypomethylated genome, hyperacetylated histones and increased levels of chromatin accessibility.^3^, ^27^ Similarly, experimental perturbations lead global chromatin changes, including treatment with histone deacetylase (HDAC) inhibitors, knock‐down of the chromatin assembly factors (such as *Caf‐1, G9a, Hp1, Kap‐1* or *Kdm1a*) all trigger 2C‐like state conversion.^16^, ^28^, ^29^ However, acute DNA demethylation is not sufficient for MERVL network activation of 2C‐like cells in S/L condition.^27^ Here, we sought to investigate whether the RA‐induced 2C‐like state is associated with the global DNA hypomethylation. For this, we supplemented a well‐known DNA methyltransferase inhibitor 5‐aza‐2'‐deoxycytidine (5‐Aza)^30^, ^31^ into 2i/L‐ESCs and RA‐2i/L‐ESCs culture medium. We found 5‐Aza treatment is unable to induce NELFA‐mediated 2C‐like state activation in 2i/L medium, even if three days of continuous treatment (Figure 4A,B). This result is consistent with the previous result that acute DNA demethylation is not sufficient for MERVL network activation of 2C‐like cells.^27^ However, 5‐Aza treatment in RA‐2i/L‐ESCs group significantly increases NELFA‐positive 2C‐like cells up to 10.2% (Figure 4A,B). Our data demonstrate a synergistic effect of 5‐Aza and RA on activating NELFA derived 2C‐like state of ESCs cultured in N2B27 based 2i/L medium. Meanwhile, the expression levels of DNA methylation‐related genes (such as *Dnmt1, Dnmt3l* and *Uhrf1*) were downregulated in RA‐2i/L‐ESCs compared with 2i/L‐ESCs (Figure 4C). Notably, the protein level of DNMT3A, DNMT3L and DNMT1 was also downregulated in RA‐2i/L‐ESCs compared with 2i/L‐ESCs (Figure 4D). This result is consistent with previous report showing that *Dnmt1* impedes activation of 2C genes in 2C‐like cells transition.^32^ However, the exact regulatory mechanism of how RA treatment induces DNA demethylation and promotes 2C‐like state of ESCs via NELFA requires further investigation.

2‐cell embryo‐like cells also display high core‐histone mobility, suggesting that histone mobility may be linked to greater cellular plasticity.^16^, ^33^ For example, 2C‐like cells display higher levels of the ‘active’ marks on H3K4me2, pan‐acetylated H3 and pan‐acetylated H4 compared with ESCs.^3^, ^16^ Therefore, we wondered whether RA treatment can alter histone modifications landscapes that are associated with 2C‐like cells. Transcriptional analysis showed that majority histone deacetylation related genes were highly expressed in 2i/L‐ESCs compared with RA‐2i/L‐ESCs (Figure 4E). Expectedly, ‘active’ histone mark H3K27 acetylation (H3K27ac) level was significantly increased in RA‐2i/L‐ESCs compared with 2i/L‐ESCs (Figure 4F). Thus, retinoic acid‐induced 2C‐like state of ESCs mainly associated with epigenetic modifications such as DNA demethylation and histone acetylation.

### 3.5 Retinoic acid regulates 2C‐like state by metabolic process

**FIGURE 5:**
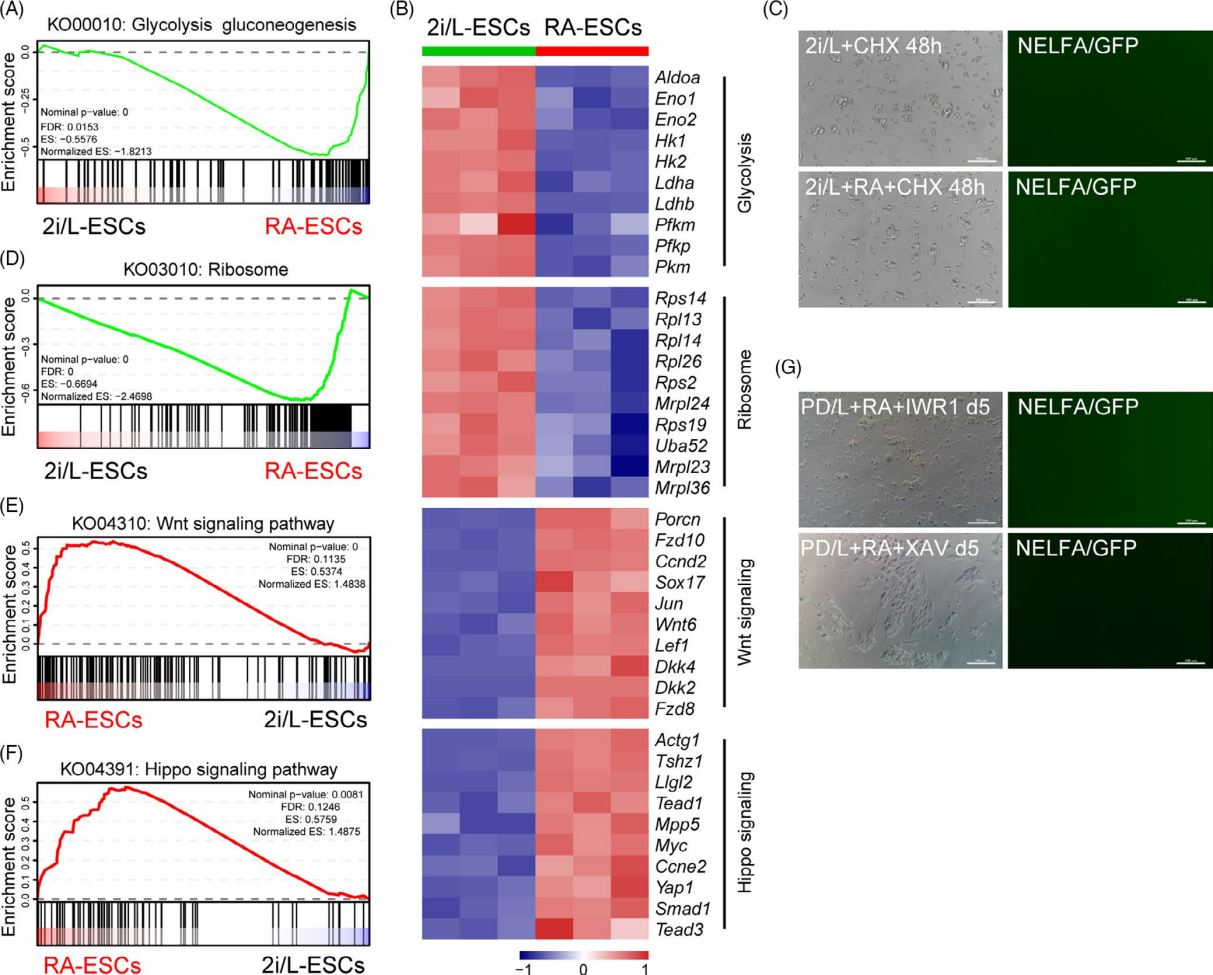
Retinoic acid regulates 2C‐like state by metabolic process. A, RA‐2i/L‐ESCs show global downregulation of glycolysis gluconeogenesis‐related genes by gene set enrichment analysis (GSEA). Normalized enrichment score (NES) and nominal *P* values are shown. B, Heat map showing expression levels of metabolic process and signalling pathway genes in 2i/L‐ESCs and RA‐2i/L‐ESCs. C, NELFA‐GFP reporter 2i/L‐ESCs and RA‐2i/L‐ESCs were treated with protein synthesis inhibitor cycloheximide (CHX). Scale bars, 100 μm. D, RA‐2i/L‐ESCs show global downregulation of ribosome‐related genes by gene set enrichment analysis (GSEA). Normalized enrichment score (NES) and nominal *P* values are shown. E, RA‐2i/L‐ESCs show global upregulation of WNT signalling pathway‐related genes by gene set enrichment analysis (GSEA). Normalized enrichment score (NES) and nominal *P* values are shown. F, RA‐2i/L‐ESCs show global upregulation of Hippo signalling pathway‐related genes by gene set enrichment analysis (GSEA). Normalized enrichment score (NES) and nominal *P* values are shown. G, RA‐2i/L‐ESCs were removed CHIR99021 and cultured with WNT signalling inhibitor IWR1 or XAV939 containing medium. Scale bars, 100 μm

Several studies have documented metabolic differences between 2C‐like cells and ESCs, and specifically, 2C‐like cells exhibit decreased glycolytic, respiratory activity and lower levels of reactive oxygen species.^10^, ^34^, ^35^ Here, we wondered if the metabolic features of 2C‐like cells in RA treatment might be altered. To uncover the role of RA in metabolic processes, we performed gene set enrichment analysis (GSEA) against the KEGG database and gene ontology (GO) analysis for 2i/L‐ESCs and RA‐2i/L‐ESCs transcripts. As expected, glycolysis gluconeogenesis (KO00010), monosaccharide catabolic (GO0046365), pyruvate metabolism (KO00620) and oxidative phosphorylation (KO00190) are significantly reduced in RA‐2i/L‐ESCs compared with 2i/L‐ESCs (Figure 5A and Figure S3A). In particular, we note that the glycolysis pathway‐related genes (such as *Hk1, Hk2, Pfkm* and *Pfkp*) were significantly downregulated in RA‐2i/L‐ESCs (Figure 5B). In addition, inhibition of glycolysis in S/2i/L condition can induce 2C‐like state, but not in N2B27‐based medium (Figure S1A). This is congruous with our previous study where treatment with 2‐deoxy‐D‐glucose (2‐DG), a glycolysis inhibitor, significantly increases NELFA‐positive cells in S/2i/L condition (Figure S3B).^10^ Our results suggest that RA regulates 2C‐like cells through suppressing glycose metabolism in N2B27‐based medium and partially substitute the function of serum and induce NELFA.

It has been reported that there are high protein turnover rates in MERVL^+^ Zscan4^+^ cells.^3^, ^16^ We thus wondered if the inhibition of protein synthesis can induce 2C‐like cells in vitro culture. We found that supplementing cycloheximide (CHX), protein synthesis inhibitor, 2i/L‐ESCs and RA‐2i/L‐ESCs failed to activate 2C‐like cells (Figure 5C). This suggests that general protein depletion in MERVL^+^ Zscan4^+^ cells may occurs after the 2C activation. In line with this, the site of biological protein synthesis, ribosome (KO03010 and GO0005840) and ribosomal subunit (GO0015934 and GO0015935) related genes expression levels in RA‐2i/L‐ESCs were significantly reduced compared to 2i/L‐ESCs (Figure 5B,D and Figure S3C).

Embryonic stem cells pluripotency is primarily regulated by canonical WNT/β‐CATENIN, FGF/ERK and JAK/STAT3 signalling.^36^, ^37^, ^38^, ^39^, ^40^ Interestingly, expression levels of WNT signalling pathway (KO04310) and Hippo signalling pathway (KO04391) related genes were significantly upregulated in RA‐2i/L‐ESCs when compared to 2i/L‐ESCs (Figure 5B,E,F). In addition, the protein levels of β‐CATENIN are slightly increased in RA‐2i/L‐ESCs compared to 2i/L‐ESCs (Figure 4D). To investigate the role of WNT/β‐CATENIN signalling in RA‐2i/L‐ESCs, we removed CHIR99021 from the culture medium and supplemented with either IWR1 or XAV939 WNT signalling inhibitor. We found that once WNT signalling is suppressed, RA‐2i/L‐ESCs are unable to maintain pluripotency and rapidly differentiates (Figure 5G), indicating that RA‐2i/L‐ESCs rely on WNT/β‐CATENIN signalling for self‐renewal and maintenance of pluripotency network similar to 2i/L‐ESCs (Figure S3D). In sum, we showed that metabolic processes of 2C‐like cells are different from ESCs.

## 4 DISCUSSION

**FIGURE 6:**
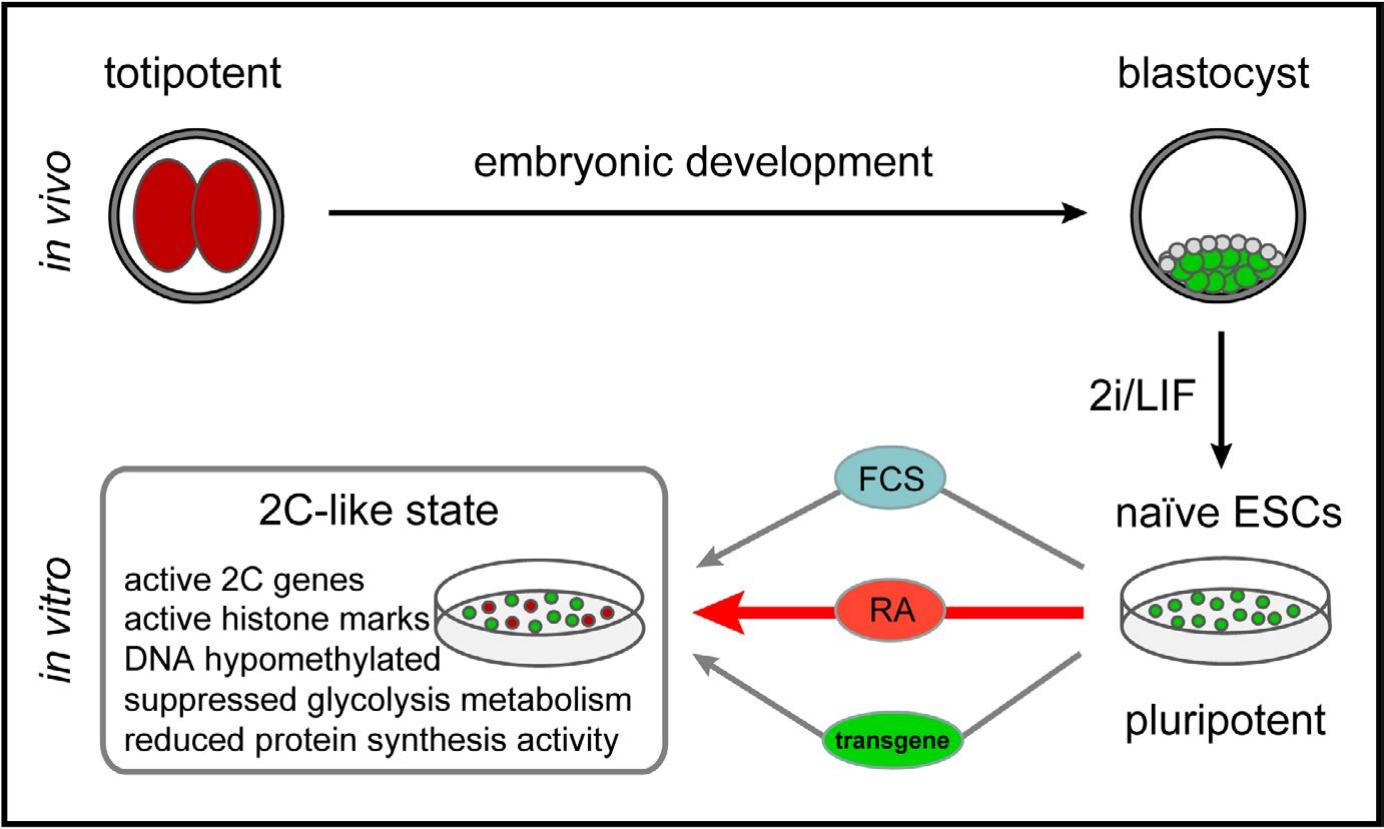
Retinoic acid induces NELFA‐mediated 2C‐like state of ESCs in chemically defined media. 2C‐like cells arise spontaneously in ESCs upon serum and leukaemia inhibitory factor (LIF) containing culture condition and some genetic modified ESCs, and therefore, 2C‐like cells refer to totipotent. Intriguingly, naïve ESCs culture medium fail to induce 2C‐like state of ESCs. In this study, we report a robust in vitro induction system, based on retinoic acid (RA) containing chemically defined media, which can efficiently increase 2C‐like cells population in culture without any genetic modification and serum

Retinoic acid is member of the nuclear receptor superfamily that is discovered in 1987 as receptor for vitamin A metabolite.^41^, ^42^ The action of RA hinges on nuclear receptors, a family of ligand‐regulated transcription factors that control a wide range of developmental processes. Recently, RA‐dependent molecular mechanism for the maintenance and differentiation of ESCs and especially, for entry into 2C‐like state has been reported.^11^, ^19^, ^20^, ^43^, ^44^ 2C‐like transition is induced by serum containing media or short‐time culture in supplement with RA, but the defined function is remaining unclear. Here, to fill in this knowledge gap, we screened chemical compounds and identified relevant genetic and epigenetic regulation. We report a robust in vitro culture system based on RA containing chemically defined media which can efficiently increase 2C‐like cells, and stably maintain the 2C‐like state (Figure 6). Interestingly, prolonged retinoic acid induction did not affect ESCs pluripotency networks and is able to induce MERVL or/and NELFA‐mediated 2C‐like state activation. Notably, the 2C‐like cells obtained in chemically defined medium can contribute to both ICM and TE in vivo.

In this study, we used two different 2C reporter cell lines, MERVL::tdTomato and NELFA‐GFP, to measure 2C induction following RA treatment. We found that RA treatment resulted in high expression of 2C‐specific genes in both reporter cells. The results showed RA has broader role in 2C‐like cells transition of ESCs. In addition, we observed that when 5‐Aza, inhibitor of DNA methylation, is added in 2i/L+RA and control 2i/L group, NELFA‐positive cells are increased in 2i/L+RA group (10.2%) compare control (0.06%). Our findings support that 2C‐like cells exhibit DNA hypomethylation state^27^ and also enhance 2C‐like cells by inhibitor of DNA methylation, and however, only 5‐Aza treatment failed continues to keep high percentage of NELFA‐positive cells in high passage (p16) RA‐2i/L‐ESCs (Figure S3E). Notably, the level of ‘active’ mark H3K27 acetylation in ESCs are increased after RA and RA+5aza (Figure 4F), and this is consistent with previous report.^20^ Here, we also showed that reducing the expressions of *Dnmt1*, *Dnmt3l* and *Uhrf1* in RA‐2i/L‐ESCs, which also in line with previous studies.^32^ They indicated that *Dnmt1* serves as a barrier mainly during 2C genes activation and the MERVL‐LTR transcriptional network is not significantly upregulated in DNMT triple‐knockout (TKO) ESCs.^27^, ^32^, ^45^ However, they used S/L culture condition, it is difficult to determine which factors in serum play an important role in 2C‐like cells regulation. In our study, retinoic acid activates 2C‐like state in 2i/L medium to provide a new way to study 2C‐like transition in ESCs.

In addition, except for transcriptome and epigenome‐related features, 2C‐like cells exhibit some distinctive metabolic features, and they display reduced oxygen consumption rates than do ESCs, which similar to early‐stage embryos also display lower oxygen consumption compared with blastocysts.^34^ In our study, RA‐2i/L‐ESCs have low glycolysis gluconeogenesis and low oxidative phosphorylation level and similar to our previous study and other reports.^10^, ^34^ Notably, in chemical‐defined condition, 2‐DG could not induce NELFA expression, which different with S/L condition.^10^ We hypothesis 2i/L medium supplemented with RA provide 2C‐like gene expression stable and plateau in ESCs. In conclusion, our study not only reveals retinoic acid induces ESCs to 2C‐like transition by NELFA in defined culture condition, but also demonstrates that retinoic acid mediates 2C‐like state by epigenetic and metabolic regulation.

## CONFLICT OF INTEREST

The authors declared no potential conflicts of interest.

## AUTHOR CONTRIBUTIONS

BW, SB and XL designed the experiments. YW, BW and QN conducted the experiments and analysed the data. BW and YW prepared the manuscript and figures with help from all the authors. WT helped to proof the manuscript and provide the NELFA‐Strep‐HA‐P2A EGFP reporter and mCherry‐labelled ESCs.

## Supporting information

Fig S1

Fig S2

Fig S3

Table S1

## Data Availability

These RNA‐seq data are available through the NCBI Sequence Read Archive (SRA) under the ID PRJNA677836 (https://dataview.ncbi.nlm.nih.gov/object/PRJNA677836?reviewer=j7qe9trpm8kkfdietmm38upg26). Data of pre‐implantation embryos (E1.5, E2.5 and E3.5) are from previously published data (GSE126056 and GSE66582).^25^, ^26^ All data that support the conclusions in the study are available from the authors on reasonable request.
