# Supplementary figures and images for: Retinoic acid induces NELFA‐mediated 2C‐like state of mouse embryonic stem cells associates with epigenetic modifications and metabolic processes in chemically defined media
Source: Cell Prolif. 2021 May 7;54(6):e13049. doi: 10.1111/cpr.13049 (PMC8168409; doi:10.1111/cpr.13049)

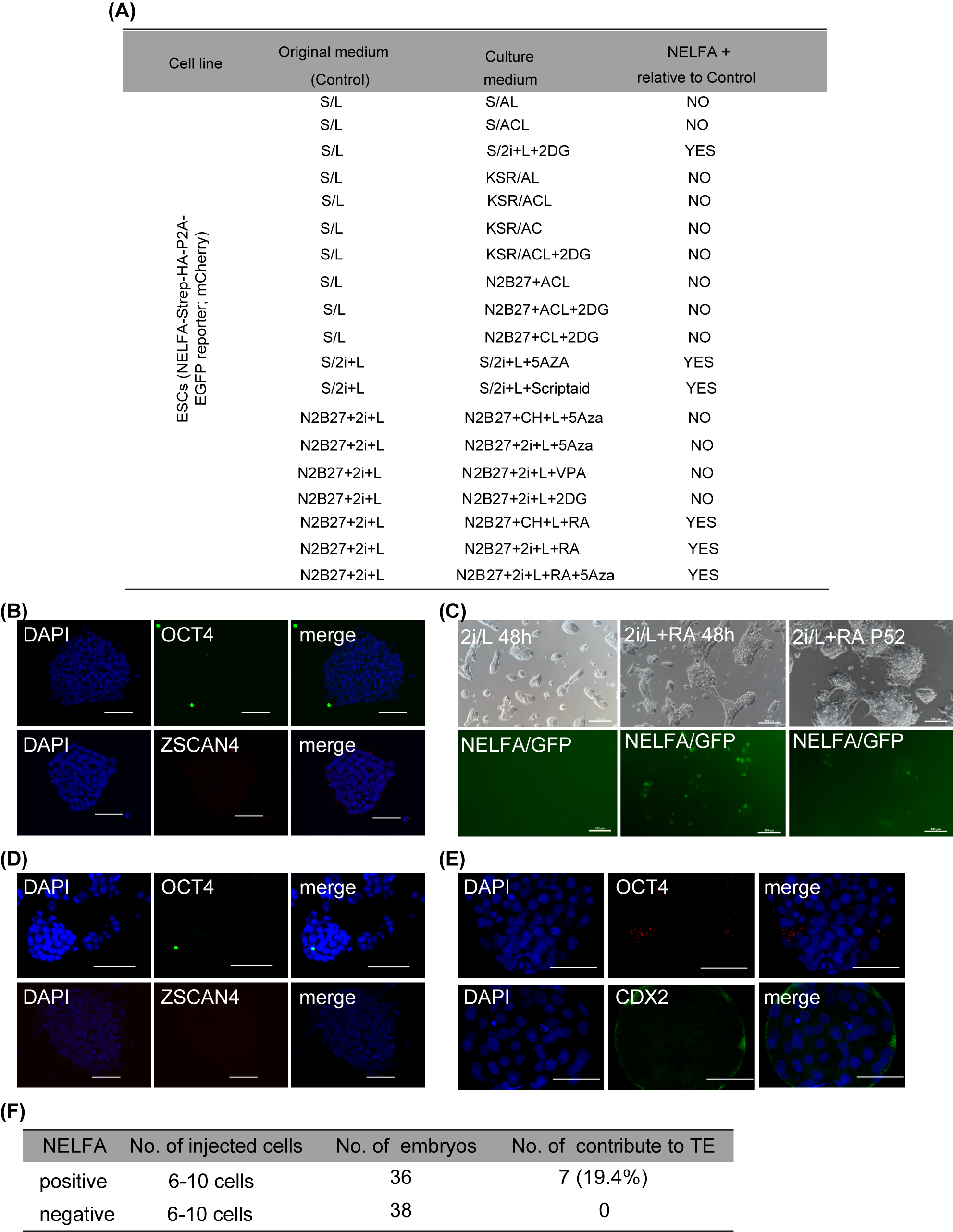

Supplement: Supplementary file 1 — Fig S1 [file CPR-54-e13049-s004.tif]

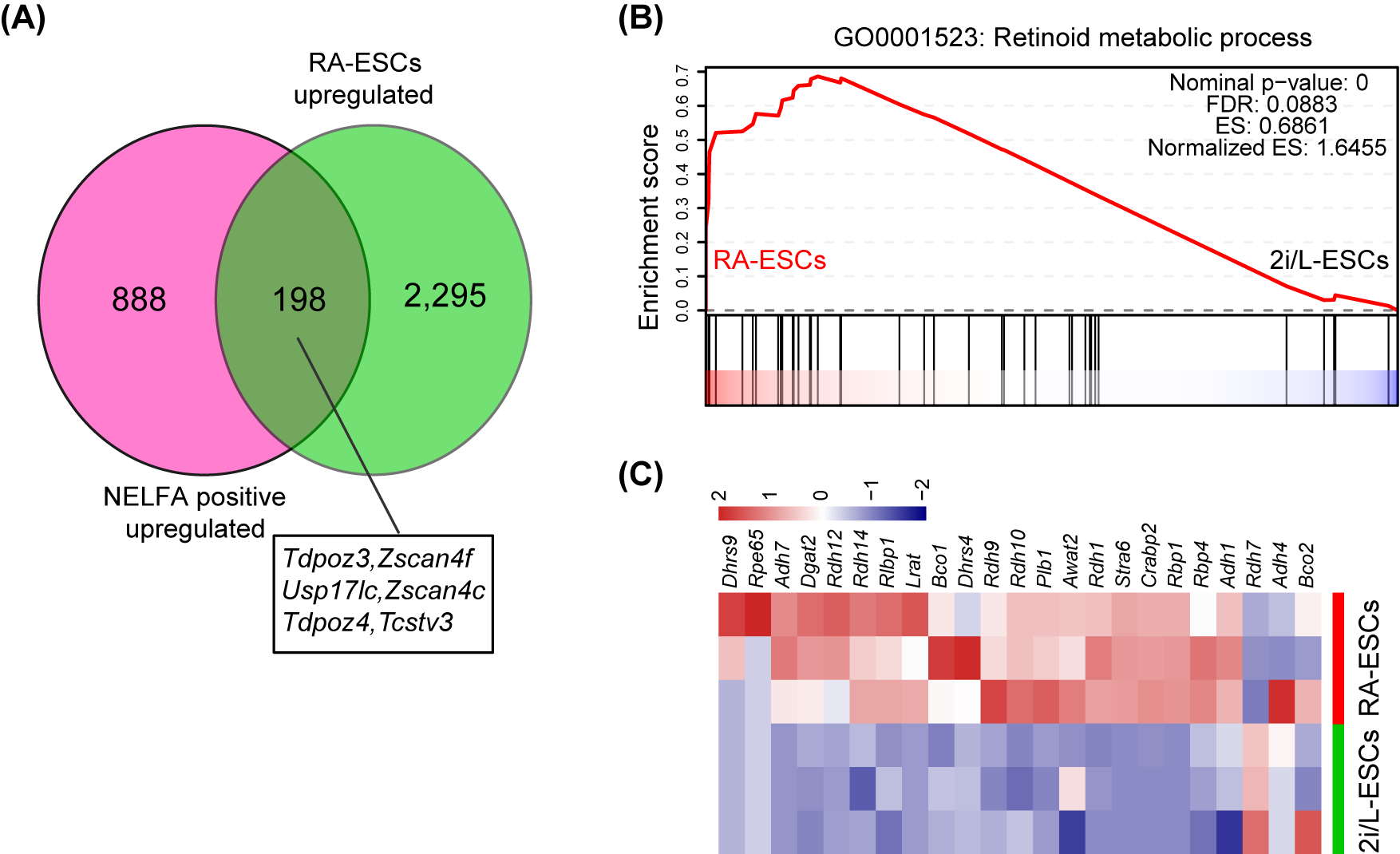

Supplement: Supplementary file 2 — Fig S2 [file CPR-54-e13049-s003.tif]

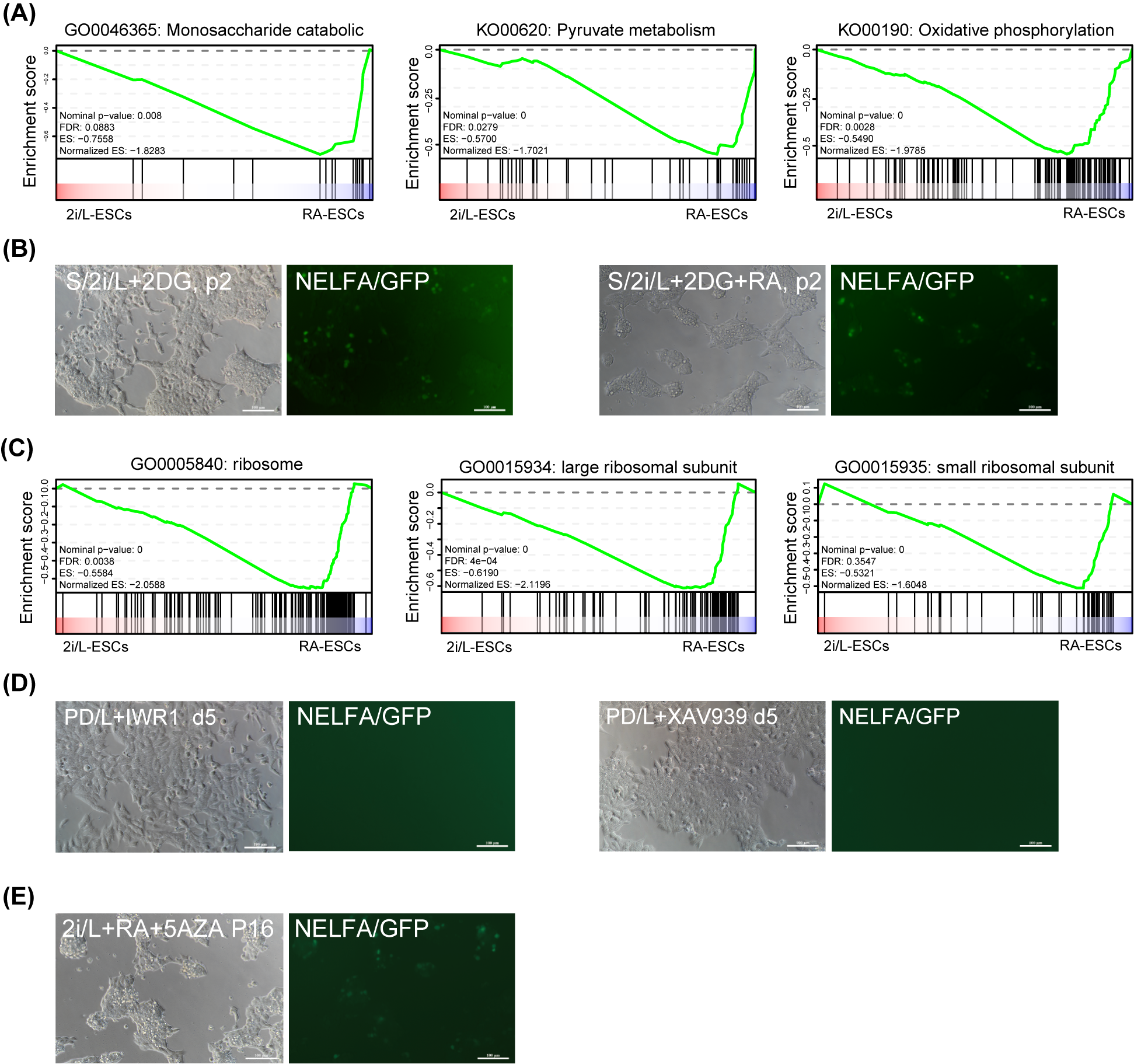

Supplement: Supplementary file 3 — Fig S3 [file CPR-54-e13049-s001.tif]
